# A High Protein Diet during Pregnancy Affects Hepatic Gene Expression of Energy Sensing Pathways along Ontogenesis in a Porcine Model

**DOI:** 10.1371/journal.pone.0021691

**Published:** 2011-07-18

**Authors:** Michael Oster, Eduard Murani, Cornelia C. Metges, Siriluck Ponsuksili, Klaus Wimmers

**Affiliations:** 1 Research Unit Molecular Biology, Leibniz Institute for Farm Animal Biology (FBN), Dummerstorf, Germany; 2 Research Unit Physiology of Nutrition, Leibniz Institute for Farm Animal Biology (FBN), Dummerstorf, Germany; 3 Research Group Functional Genomics, Leibniz Institute for Farm Animal Biology (FBN), Dummerstorf, Germany; Ecole Normale Supérieure de Lyon, France

## Abstract

In rodent models and in humans the impact of gestational diets on the offspring's phenotype was shown experimentally and epidemiologically. The underlying programming of fetal development was shown to be associated with an increased risk of degenerative diseases in adulthood, including the metabolic syndrome. There are clues that diet-dependent modifications of the metabolism during fetal life can persist until adulthood. This leads to the hypothesis that the offspring's transcriptomes show short-term and long-term changes depending on the maternal diet. To this end pregnant German landrace gilts were fed either a high protein diet (HP, 30% CP) or an adequate protein diet (AP, 12% CP) throughout pregnancy. Hepatic transcriptome profiles of the offspring were analyzed at prenatal (94 dpc) and postnatal stages (1, 28, 188 dpn). Depending on the gestational dietary exposure, mRNA expression levels of genes related to energy metabolism, N-metabolism, growth factor signaling pathways, lipid metabolism, nucleic acid metabolism and stress/immune response were affected either in a short-term or in a long-term manner. Gene expression profiles at fetal stage 94 dpc were almost unchanged between the diets. The gestational HP diet affected the hepatic expression profiles at prenatal and postnatal stages. The effects encompassed a modulation of the genome in terms of an altered responsiveness of energy and nutrient sensing pathways. Differential expression of genes related to energy production and nutrient utilization contribute to the maintenance of development and growth performance within physiological norms, however the modulation of these pathways may be accompanied by a predisposition for metabolic disturbances up to adult stages.

## Introduction

The relationship between the intrauterine adaptive response to adverse changes in the biological environment with persistent postnatal effects and permanent consequences on the phenotype is termed ‘fetal programming’. Due to developmental plasticity, interactions between genotype and environment lead to adaptations in gene expression and ultimately to a modified phenotype [1]. In various human and animal studies it was shown that low maternal protein intake during pregnancy is associated to low birth weight, and subsequently to disorders of metabolic health as well as body composition of the offspring [2]–[5]. Interestingly, it has been demonstrated that a low protein diet fed to pregnant dams will lead to intrauterine growth retardation (IUGR) and reduced birth weight [6]–[10]. Moreover, there are cues that diet-dependent modifications of the metabolism induced during fetal life can persist until adulthood. These observations were summarized to generate the ‘thrifty phenotype hypothesis’ [11] suggesting that a poor fetal nutrient supply causes low birth weight and increases the risk for adult metabolic disease. Less is known about possible effects of gestational high protein diets on offspring development and later health. It has been previously shown in rats, mice, and pigs that maternal excess protein intake during pregnancy is associated to IUGR, and results suggest that this is related to net energy deficiency in the gestating dam [12]–[14]. Interestingly, epidemiological studies in women show that high protein intakes during pregnancy can also result in fetal growth retardation [15]–[17]. Here, we fed a very high protein excess (30% CP) in order to promote effects on the offspring at the molecular (transcriptome, proteome) and organismal level.

Altered circulating levels of a number of metabolically active hormones like insulin or cortisol and mitochondrial energy-producing pathways are involved in the short term regulatory and long-term adaptive processes association with nutritional programming [2], [18]–[20]. Because the liver is the central metabolic organ and its activities are essential for storage, utilization, and partitioning of nutrients it is the major target of molecular mechanisms leading to the establishment of a metabolic memory. Studies on nutritional programming were mainly done in rodent animal models and most studies were focused on biochemical and transcriptional changes of selected candidate metabolic and/or signaling pathways and their related genes at particular developmental stages. However, longitudinal holistic studies of the modulation of the offspring transcriptome due to maternal dietary supply during pregnancy are scarce. In order to contribute to a comprehensive inventory of genes and functional networks that are targets of nutritional programming initiated during fetal life, we applied whole-genome microarrays for expression profiling in a longitudinal experimental design covering prenatal, perinatal, juvenile and adult ontogenetic stages. A porcine model was used, where pregnant sows were fed either a gestational high protein diet or an adequate protein diet on an isoenergetic basis to investigate the effects on hepatic gene expression in their fetuses and offspring. We focussed on four key developmental stages to gain knowledge regarding the complex regulatory mechanisms between different dietary protein levels during pregnancy and the transcriptome level of the offspring.

There are multiple factors for the development of intrauterine growth retardation, including maternal gestational diets, which are an issue in pig breeding. In this context it is of interest that lower birth weight piglets (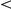
1.2 kg) later on show lower daily gains, higher body fat contents and lower muscle mass [21]. Moreover, the study comprises a valuable model for nutritional programming in humans, since pigs and humans share similarity in metabolism, physiology, anatomy and genome [22], [23]. These experimental data will complement existing findings from rodent models and epidemiological human data. In particular the high protein diet provides a model for dietary recommendations to combat obesity in human. The porcine model used here shows that the porcine offspring, which was exposed to an oversupply with protein during fetal development but had appropriate postnatal dietary conditions, was able to adapt in terms of their organismal phenotype [14], [24], [25]. In fact, in our experiment, newborns from sows that received a high protein supply during gestation had a significantly lower birth weight and in particular a lower body fat content than newborns of the control group. However, neither body weight, body composition, and cellularity of muscle and adipose tissue of weaning piglets, nor carcass weight, carcass composition, and meat quality at slaughter differed significantly among offspring of the HP and the AP group [14], [24], [25]. However, the transcriptomic analysis presented here, reveals that the hepatic expression profiles show altered responsiveness of energy sensing pathways and indicate a predisposition to metabolic disturbances up to adult stages.

## Results

We performed a longitudinal holistic study of the hepatic transcriptome modulation due to maternal gestational dietary supply in order to provide a comprehensive inventory of genes and functional networks that are targets of fetal nutritional programming using a porcine model. Regarding the effect of ‘fetal programming’ pregnant sows were fed either a high protein diet (HP) containing 30% crude protein or an isocaloric adequate protein diet (AP) containing 12% crude protein. We investigated their offspring's hepatic gene expression in one prenatal and three postnatal stages with the help of porcine 24 k-microarrays. In total we found 12,477 probe-sets expressed at stage 94 dpc (1 dpn: 12,646; 28 dpn: 12,285; 188 dpn: 11,835) according to MAS5 analysis. Further filtering based on the variability of expression of probe-sets revealed 7,802 probe-sets for further analysis at stage 94 dpc (1 dpn: 10,253; 28 dpn: 7,971; 188 dpn: 8,950). These probes sets represent 5,887 genes at stage 94 dpc (1 dpn: 7,699; 28 dpn: 6,194; 188 dpn: 6,903) according to the recent annotation [26]. In order to identify molecular pathways affected by the gestational diets we first analysed differential expression between the dietary groups within each stage separately. The different dietary exposure of the offspring during prenatal development can be expected to cause slight shifts of the developmental age of the offspring that may be reflected by subtle changes of the transcriptome and could hamper the identification of direct effects of the gestational diets on the hepatic expression. Secondly, we analysed the differences among both experimental groups regarding the more long-term and more pronounced changes of expression patterns between the adjacent stages. In total we found 13,459 probe-sets expressed within 94 dpc and 1 dpn (1 dpn and 28 dpn: 13,557; 28 dpn and 188 dpn: 12,629) according to MAS5 analysis. After the filtering steps described above, 10,462 probe-sets were detected within 94 dpc and 1 dpn (1 dpn and 28 dpn: 10,636; 28 dpn and 188 dpn: 8,686). These probes sets represent 7,817 genes within 94 dpc and 1 dpn (1 dpn and 28 dpn: 7,940; 28 dpn and 188 dpn: 6,704). Notably, q-values between ontogenetic stages within diet were remarkable lower (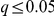) than between diets within stage (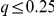).

### Comparisons between HP and AP within stages

**Figure 1:**
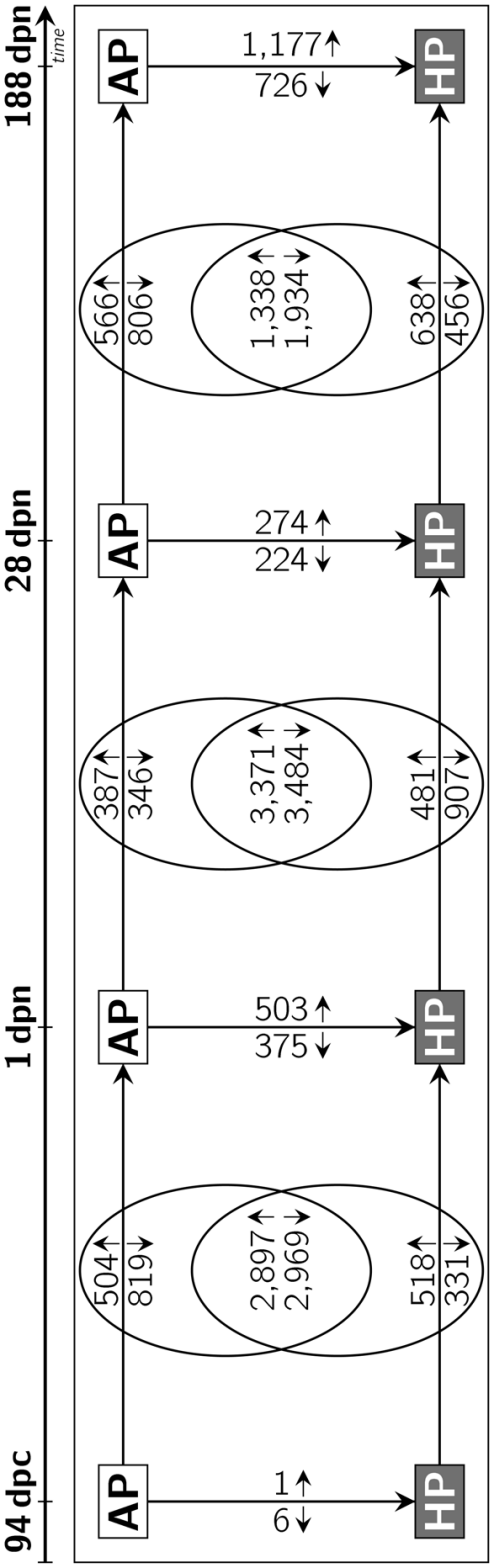
Number of regulated probe-sets in liver tissue. The numbers at the horizontal arrows indicate the quantity of probe-sets significantly regulated between the adjacent ontogenetic stages in either AP or HP offspring, whereas the numbers in the intersections indicate the quantity of probe-sets commonly regulated between stages in AP and HP offspring. The numbers at vertical arrows are the number of probe-sets differentially expressed between AP and HP offspring at the same ontogenetic stage (arrows between boxes show direction of the comparisons; small arrows to top = up-regulated, small arrows to bottom = down-regulated probe-sets).

Expression of mRNA was compared in HP and AP offspring within each ontogenetic stage (Figure 1). At stage 94 dpc 7 probe-sets differed significantly between HP and AP fetuses (1 increased). It was not possible to assign those genes to significant regulated metabolic pathways. In perinatal piglets (stage 1 dpn) 878 probe-sets differed between HP offspring (503 for HP
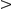
AP) and AP offspring. Ingenuity Pathway Analysis indicates enrichment of molecular routes related to energy metabolism, lipid metabolism, N-metabolism, cellular growth and immune response (Table 1). In particular, genes associated with oxidative phosphorylation (OXPHOS), biosynthesis of steroids, and valine, leucine and isoleucine degradation were found diminished in HP offspring. Furthermore, genes associated with RAN signaling as well as PPARGC1a and PRKAA2 showed an increased expression in HP offspring at stage 1 dpn. Analysis towards common regulation of genes higher expressed in HP than AP revealed 217 potential regulatory elements (RE) (177 for HP
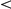
AP) that corresponded to 103 transcription factors (TF) (107 for HP
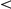
AP). In juvenile piglets (stage 28 dpn) 498 probe-sets differed between HP and AP offspring. The expression of 274 probe-sets was increased in the HP offspring compared with AP offspring. At juvenile stage, pathways of lipid metabolism and N-metabolism were enriched. Genes associated with fatty acid metabolism, and valine, leucine and isoleucine degradation showed a decreased mRNA expression in HP offspring. Analysis using DiRE identified 125 RE within the genes up-regulated in HP (103 for HP
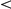
AP) that were associated with 101 TF (100 for HP
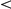
AP). In adult pigs (stage 188 dpn) 1,903 probe-sets were significantly different between HP and AP offspring. Of these 1,177 probe-sets showed higher expression and 726 probe-sets showed lower expression in HP than in AP. The mRNA expression levels of genes associated with glucocorticoid receptor signaling, RAN signaling, PPAR signaling and IGF-1 signaling as well as fatty acid elongation in mitochondria and mitochondrial dysfunction were increased in HP offspring. The genes up-regulated in HP compared to AP were related to 117 (118 for HP
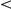
AP) TF that fitted 448 potential RE (190 for HP
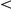
AP). No genes were found consistently differentially expressed between the groups along all examined stages. However, at 1 dpn and 188 dpn 142 probe-sets were differentially regulated in both stages between HP and AP.

**Table 1:** Significantly regulated transcripts of metabolic pathways in liver tissue within different ontogenetic stages (Ingenuity Pathway Analysis).

Ontogenetic stage	Regulated pathway	Regulation	*P* value	No. of regulated genes	Genes involved in pathway
94 dpc	-	-	-	-	-
1 dpn	RAN signaling	up	4.57*E-7	6	IPO5, KPNA3, KPNB1, RAN, RANBP2, TNPO1
	Oxidative phosphorylation	down	2.01*E-15	23	ATP5H, ATP5I, ATP5L, ATP6V0D1, COX6A1, COX6B1, COX6B2, COX7A2, COX7B, COX8A, NDUFA3, NDUFA4, NDUFA13, NDUFB1, NDUFB5, NDUFB6, NDUFB7, NDUFB11, NDUFS7, NDUFS8, SDHB, TCIRG1, UQCR11
	Biosynthesis of steroids	down	1.19*E-10	10	DHCR7, EBP, FDFT1, FDPS, HMGCR, IDI1, MVK, NQO2, PMVK, SQLE
	Val, Leu, Ile degradation	down	3.41*E-2	4	ACAA1, ACAA2, ACAT2, HMGCS1
28 dpn	Val Leu, Ile degradation	down	6.50*E-4	5	ALDH1A1, ALDH7A1, HADHA, HMGCS1, IVD
	Fatty acid metabolism	down	9.55*E-3	5	ACOX1, ALDH1A1, ALDH7A1, HADHA, IVD
188 dpn	Glucocorticoid receptor signaling	up	1.75*10-3	25	A2M, AKT3, CD163, FOS, GRB2, GTF2A2, GTF2E2, GTF2F2, HSP90AA1, HSPA8, HSPA9, ICAM1, IL10, JAK1, MAP2K1, MAPK1, MAPK14, PPP3CB, RRAS2, STAT5B, SUMO1, TAF4, TAF7, TAF15, TGFBR2
	RAN signaling	up	8.21*10-3	4	IPO5, KPNA1, KPNA6, RAN
	IGF-1 signaling	up	3.62*10-3	12	AKT3, CSNK2A1, FOS, GRB2, IGF1, MAP2K1, MAPK1, PRKAR1A, RRAS2, YWHAG, YWHAQ, YWHAZ
	PPAR signaling	up	3.82*10-2	9	FOS, GRB2, HSP90AA1, IL18, MAP2K1, MAP4K4, MAPK1, RRAS2, STAT5B
	Fatty acid elongation in mitochondria	up	1.42*10-3	5	ACAA2, HADHB, HSD17B4, PECR, PPT1
	Mitochondrial dysfunction	up	3.63*10-3	12	AIFM1, APP, CYCS, GLRX2, MAOA, NDUFB3, NDUFB6, NDUFS1, PDHA1, PSEN1, SOD2, UQCRB

The comparison between the dietary gestational protein levels (HP vs. AP) is shown in dependence of the regulatory direction (up or down).

### Differences of longitudinal ontogenetic regulation among HP and AP offspring

**Figure 2:**
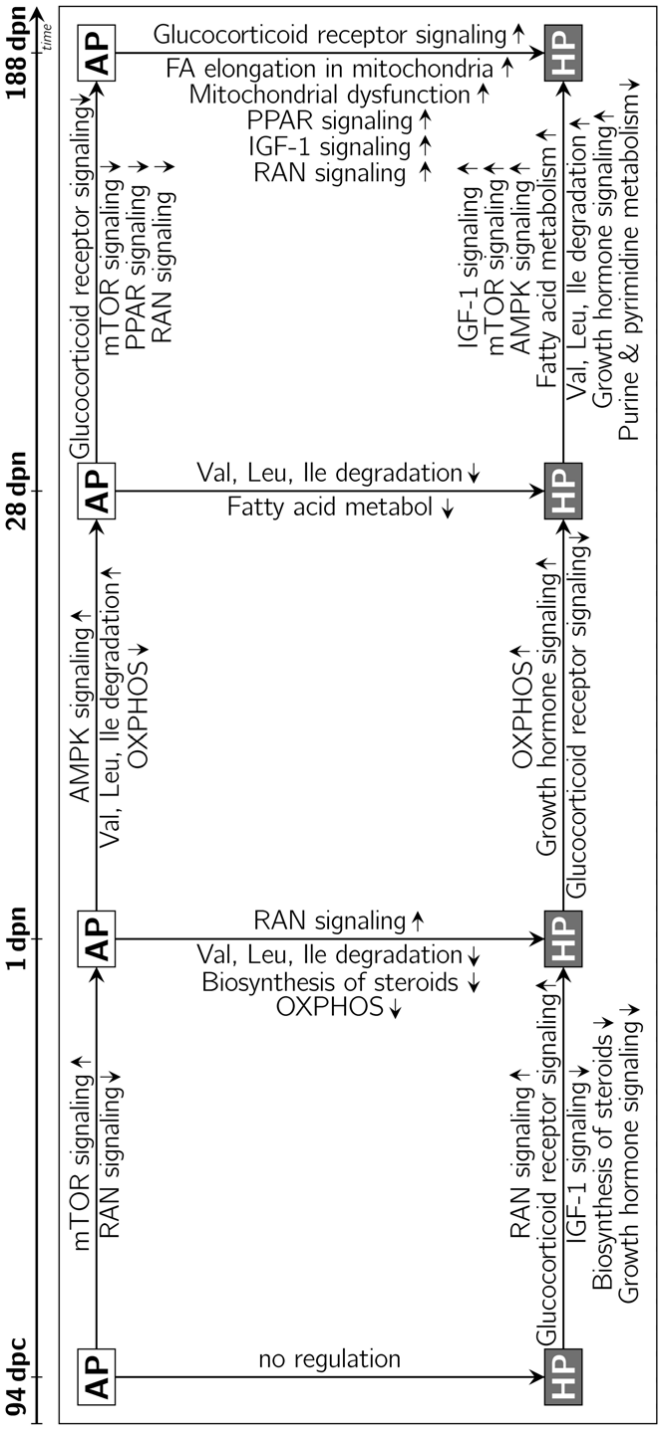
Regulated pathways in liver tissue between ontogenetic stages and maternal diets. Listed pathways between stages in AP offspring (white boxes) indicate the appropriate ontogenetic development, which doesn't occur in HP offspring (black boxes) at the corresponding developmental period. Pathways between the HP stages indicate processes and metabolic regulations, which occur in the HP offspring but not in the AP offspring in the corresponding developmental period. The differences in gene regulation dependent on diet and ontogenetic stage indicate fetal programming in terms of developmental and metabolic disorders (arrows between boxes show direction of the comparisons; small arrows to top = up-regulated, small arrows to bottom = down-regulated pathways; mTOR, mammalian target of rapamycin; RAN, Ras-related nuclear protein; IGF-1, insulin-like growth factor; PPAR, peroxisome proliferator receptor; AMPK, AMP-activated kinase).

Considering two adjacent ontogenetic stages within one treatment group, significantly regulated transcripts were determined. The resulting gene lists were compared between HP and AP offspring at the corresponding ontogenetic periods. The intersection of commonly regulated genes between those comparisons was discarded because these regulations were likely due to physiologically developmental processes. Consequently only genes, whose regulation between two consecutive ontogenetic stages (period I: 94 dpc-1 dpn; period II: 1 dpn–28 dpn; period III: 28 dpn–188 dpn) was private to either the HP or the AP group were analysed. These genes display diet-dependent longitudinal transcriptomic regulation (Figure 1). Thus, genes and pathways identified as regulated in one offspring group were either unregulated or showed an opposite direction of regulation in the corresponding ontogenetic comparison within the other dietary group. Between fetal and perinatal stages (period I), there were 1,323 (504 for 1 dpn
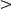
94 dpc in AP) probe-sets showing levels and directions of regulation in the AP group that were different from the HP group. The mTOR signaling was found to be increased at stage 1 dpn while genes associated with RAN signaling were decreased (Table 2). In HP offspring 849 probe-sets showed ontogenetic regulation (518 for 1 dpn
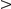
94 dpc in HP) during the corresponding time period that is specific when compared to the AP group. The mRNA expression level of genes associated with glucocorticoid receptor signaling and RAN signaling in HP offspring was increased. Some genes participating in IGF-1 signaling, biosynthesis of steroids and growth hormone signaling were found to be decreased in HP perinatal piglets. DiRE pointed to 201 potential RE matching 106 TF for the genes that are up-regulated at 1 dpn compared to 94 dpc in AP (292 RE with 110 TF for 1 dpn
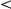
94 dpc in AP; 217 RE with 97 TF for 1 dpn
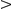
94 dpc in HP, 136 RE with 91 TF for 1 dpn
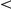
94 dpc in HP). Comparing perinatal and juvenile piglets (period II) 733 probe-sets were regulated in a different manner in AP offspring than in HP offspring. Of these, 387 probe-sets showed up regulation and 346 probe-sets showed lower expression at higher age. Expression values of genes participating in AMPK signaling and the degradation of branched chain amino acids were increased, whereas genes associated with oxidative phosphorylation were decreased in AP offspring. In the same period 1,388 probe-sets exhibited ontogenetic regulation that was specific to HP offspring. Of these, 481 probe-sets showed an increased whereas 907 probe-sets showed a decreased mRNA expression. Genes involved in oxidative phosphorylation and growth hormone signaling were increased in HP offspring, while glucocorticoid receptor signaling was found to be down regulated. Those genes that were higher expressed at 28 dpn compared to 1 dpn in the AP group only exhibited 165 potential RE that were associated with 99 TF as revealed by in-silico analysis (175 RE with 107 TF for 28 dpn
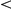
1 dpn in AP; 217 RE with 118 TF for 28 dpn
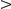
1 dpn in HP, 305 RE with 93 TF for 28 dpn
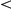
1 dpn in HP). When juvenile and young adult pigs (period III) are compared 1,372 probe-sets differed significantly (566 increased) in AP offspring. Genes higher expressed in older animals had 183 RE for 117 TF (254 RE with 103 TF for 188 dpn
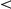
28 dpn in AP). Genes participating in glucocorticoid receptor signaling, RAN signaling, mTOR signaling and PPAR signaling were found to be down regulated. In HP offspring 1,094 probe-sets were differently expressed (638 for 188 dpn
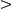
28 dpn in HP). In HP offspring genes involved in signaling pathways of IGF-1, AMPK, growth hormone and mTOR were up regulated as well as genes associated with fatty acid metabolism and degradation of branched chain amino acids. These genes covered 219 RE associated with 99 TF (160 RE with 105 TF for 188 dpn
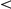
28 dpn in HP). Furthermore, genes involved in purine and pyrimidine metabolism were found to be down regulated. Figure 2 gives a comprehensive overview about the pathways found regulated between stages and diets.

**Table 2:** Significantly regulated transcripts of metabolic pathways in liver tissue between two ontogenetic stages within one dietary group (Ingenuity Pathway Analysis).

Ontogenetic comparison	Diet	Regulated pathway	Regulation	No. of regulated genes	Genes involved in pathway	*P* value
94 dpc vs. 1 dpn	AP	mTOR signaling	up	3.42*E-3	9	EIF3I, GNB1L, INSR, MRAS, PRKAA1, PRKCB, RHOG, RHOU, VEGFB
	AP	RAN signaling	down	1.43*E-2	3	KPNA6 RANBP2 TNPO1
	HP	Glucocorticoid receptor signaling	up	4.44*E-3	13	A2M, ERCC3, ESR1, GTF2H3, HMGB1, HSPA1B, MAPK9, PIK3C2A, TAF2, PIK3R1, PIK3R3, PRKACB, PTGES3
	HP	RAN signaling	up	4.93*E-3	3	CSE1L, KPNA3, TNPO1
	HP	IGF-1 signaling	down	2.13*E-3	6	FOS, IGFBP3, JUN, PIK3C2B, YWHAB, YWHAE
	HP	Biosynthesis of steroids	down	1.02*E-2	3	DHCR7, MVK, PMVK
	HP	Growth hormone signaling	down	1.33*E-2	4	FOS, IGFBP3, PIK3C2B, SOCS4
1 dpn vs. 28 dpn	AP	AMPK Signaling	up	1.89*E-4	9	AK1, AK7, AKT3, HMGCR, IRS1, MAPK14, PRKAA2, PRKAB2, PRKAG2
	AP	Val, Leu, Ile degradation	up	3.87*E-3	5	ABAT, ACAT1, BCKDHA, HMGCS1, IVD
	AP	Oxidative phosphorylation	down	2.19*E-3	8	ATP6V0E2, ATP7B, COX17, COX10, NDUFA1, NDUFB6, NDUFS8, TCIRG1
	HP	Oxidative phosphorylation	up	3.70*E-4	11	ATP6V1D, ATP6V1F, ATP6V1H, NDUFA2, NDUFA7, NDUFB1, NDUFB7, NDUFB11, NDUFS3, NDUFS7, TCIRG1
	HP	Growth hormone signaling	up	1.24*E-2	5	FOS, IGFBP3, PIK3CG, PTPN6, STAT1
	HP	Glucocorticoid receptor signaling	down	3.12*E-2	15	CDKN1A, JAK2, KRAS, MAP3K7, MNAT1, NCOA1, NR3C1, PCK2, SMAD2, SOS2, TAF5, TAT, TBP, VCAM1
28 dpn vs. 188 dpn	AP	Glucocorticoid receptor signaling	down	1.01*E-2	17	BCL2, GTF2E2, HMGB1, HSPA2, HSPA9, HSPA14, MAP3K1, MAPK14, TBP, NCOA1, NCOR1, PIK3R1, PPP3CA, PRKACB, RAC3, STAT5B, SUMO1
	AP	mTOR signaling	down	2.97*E-2	10	EIF4E, EIF4G2, EIF4G3, PIK3R1, PPP2CA, PPP2CB, PPP2R1B, PRKCB, RAC3, RHOU
	AP	RAN Signaling	down	1.82*E-2	3	CSE1L, RAN, XPO1
	AP	PPAR signaling	down	3.69*E-2	7	CITED2, MAP4K4, NCOA1, NCOR1, PDGFRA, PPARA, STAT5B
	HP	IGF-1 signaling	up	2.56*E-5	11	AKT3, CTGF, IGF1, IGF1R, IGFBP3, IRS1, PRKAG2, PRKCZ, PTPN11, RRAS2, SOS2
	HP	mTOR signaling	up	2.73*E-6	15	AKT3, DDIT4, EIF3E, EIF4A2, EIF4B, FNBP1, IRS1, PPP2R5A, PRKAB2, PRKAG2, PRKCZ, RND3, RRAS2, STK11, VEGFA
	HP	AMPK signaling	up	6.87*E-3	9	AKT3, IRS1, PPM1A, PPM1D, PPP2R5A, PRKAB2, PRKAG2, SRC, STK11
	HP	Fatty acid metabolism	up	9,37*E-3	9	ACAA1, ACADL, ACADSB, ACAT1, AUH, CYP2C18, CYP51A1, IVD, PTGR1
	HP	Val, Leu, Ile degradation	up	5.89*E-5	9	ACAA1, ACADL, ACADSB, ACAT1, AUH, DBT, HIBCH, IVD, PCCA
	HP	Growth hormone signaling	up	6.03*E-3	6	IGF1, IGF1R, IGFBP3, IRS1, PRKCZ, SOCS6
	HP	Purine metabolism	down	1.90*E-4	17	ABCC1, AK2, APRT, ATP13A2, BAT1, CANT1, MYO9B, NME1, NSF, PKM2, PNPT1, POLR1E, POLR2G, POLR3G, PRPS2, RRM1, RUVBL2
	HP	Pyrimidine metabolism	down	1.31*E-2	8	CANT1, DKC1, NME1, PNPT1, POLR1E, POLR2G, POLR3G, RRM1

The comparison between dietary gestational protein levels (AP, HP) is shown in dependence of the regulatory direction (up or down).

Among TFs found to be associated to the genes regulated due to maternal gestational diets at various developmental stages of the offspring 40 were redundantly found among the top 10 TFs highlighted by DiRE for the various comparisons made. Only HNF4A, DR1, STAT6, and TCF4 themselves were found differentially regulated due to diet and stage.

For all genes exemplarily analysed qRT-PCR confirmed the direction of differential regulation as obtained by microarray analysis. In 80% of the genes that we validated by qRT-PCR, significant expression differences in mRNA levels between the treatment groups were identified by both qRT-PCR and microarray analysis. Correlations between expression values of microarray and qRT-PCR ranged between 0.43 and 0.84 and were highly significant (Table 3). This suggests that our microarray data are reliable.

**Table 3:** Comparison of microarray data and qRT-PCR of selected transcripts.

Gene name	Microarray			qRT-PCR#			Correlation##
	p-value	FC	Regulation	p-value	FC	Regulation	Expr. values
*1 dpn*							
PPARGC1A	0.0103	+1.54	up	0.012	+1.63	up	0.51***
PRKAA2	0.0354	+1.15	up	0.007	+1.47	up	0.43**
HMGCR	0.0177	−1.53	down	0.030	−2.01	down	0.84***
DHCR7	0.0004	−1.57	down	0.001	−1.76	down	0.63***
NDUFA4	0.0081	−1.19	down	0.310	−1.15	n.r.	0.75***
NDUFS7	0.0097	−1.20	down	0.480	−1.14	n.r.	0.51***
NDUFS8	0.0223	−1.22	down	0.030	−1.32	down	0.56***
*188 dpn*							
PPARGC1A	0.9341	+1.01	n.r.	0.275	+1.22	n.r.	0.77***
PRKAA1	0.0083	−1.35	down	0.055	−1.38	down	0.74***
GADD45B	0.0766	+1.49	up	0.047	+1.81	up	0.67***

# calculated by factorial normalization on RPL10 expression values;

## p-value of Spearman's rho; n.r. - not regulated.

**p<0.01;

***p<0.001.

## Discussion

We applied whole-genome microarrays to evaluate offspring hepatic expression profiling affected by exposure to maternal isocaloric pregnancy diets with adequate or high protein concentrations. In order to investigate transcriptional features of developmental nutritional programming we conducted a longitudinal experimental design covering prenatal, perinatal, juvenile and adult ontogenetic stages in a porcine model. The comparisons of the relative mRNA abundances depending on dietary group and ontogenetic stages provide an overall view of the developmental plasticity of the liver. In the overall experimental design sampling of the heaviest and lightest offspring at 94 dpc and 1 dpn was foreseen. At these early stages the offspring depended on supply provided by the mother subjected to the experimental treatment. At later stages, 28 dpn and 188 dpn, when non-maternal, environmental effects become more relevant the animals close to the middle of the distribution for growth were used. At all stages sampling was balanced for sex. Impact of IUGR and low birth weight as well as sex on postnatal growth is well documented [27], [28], however, body weight and sex affected the expression of only a few probe-sets (Table S1). Following in utero exposure to a gestational high protein diet we could not find significant regulatory changes of metabolic pathways in porcine fetal liver; however, at the whole-body level growth restricted HP offspring was observed [14]. At this stage the fetuses are subjected to the intrauterine environment for already a long time and on the level of the transcriptome HP offspring has adapted, whereas the transcriptome of HP offspring might be significantly different at earlier fetal stages, i.e. transcriptome alterations may have preceded phenotype alterations. In perinatal piglets growth retardation was obvious among all HP offspring [14] - in the subset of animals used for the microarray analysis HP offspring showed numerically lower mean birth weight, however the differences among the HP and AP offspring failed to be significant. Anyhow, at 1 dpn the mRNA expression levels were extensively altered. At this time point the piglets are subjected to major nutritional changes that require acute adaptive regulation of the transcriptome. The adaptability to modulate expression profiles during porcine fetal development in response to a given environment seems to be quite high. This flexibility might depend on the degree of maturation and it might contribute to unchanged transcriptome levels at fetal stage. Alterations of the hepatic transcriptome profile in perinatal HP offspring may indicate stress and assign a programmed respond on new environmental cues.

The data provide a snapshot of the transcriptome, thus it remains unclear to what extent transcriptomic differences observed at pre- and perinatal stages between the experimental groups are due to chronic long-term effects or to acute stimulating event in life, like birth or weaning. Interestingly, offspring at 28 dpn showed a reduced number of differentially expressed mRNAs compared to stage 1 dpn. Also at this time point HP offspring may has adapted its expression profiles to the early postnatal feeding conditions during weaning time. However, the number of differentially expressed probe-sets increased again at adult stage. Our model indicates that gestational HP diets affected the expression profiles in a short-term as well in a long-term manner in HP offspring. The effect is characterized by an engagement of the genome that leads to different responsiveness and adaptability of the gene expression machinery to chronic and acute environmental stimuli. Due to the HP diet a number of energy sensing, producing and utilising pathways are important in these processes.

The impact of the maternal diets was obvious at the transcriptomic level in the respective offspring, though not effects on traits related to the development of the whole organism or single organs were observed at 1 dpn (in the subset of animals used), 28 and 188 dpn. This indicates that there is a direct relationship between the dietary treatments of the sows and the expression profiles of the offspring. Obviously, the modulations of the expression profiles are not consequences of impaired growth due to the experimental diets. Epigenetic effects manifesting during crucial pre- and perinatal developmental stages are regarded as mechanisms promoting nutritional (environmental) impact on the genome leading to shifted gene expression [29], [30]. Especially DNA-methylation is a mechanism of epigenetic gene regulation that might be involved [31]. Our study supports the identification of genes that are targets of epigenetic mechanisms of gene regulation. The analysis of RE and TF common to DE-genes only revealed four TF that were themselves regulated due to the dietary treatments. Together with the finding that DE-genes were mainly functionally annotated to cellular signaling pathways and metabolic pathways, this may be interpreted as an indication that the transcriptional response to prenatal nutritional challenge is primarily present at the level of these cellular signaling and metabolic routes rather than at hierarchically superior genes encoding transcription factors of regulatory networks acting at the DNA- and RNA-level.

### Oxidative phosphorylation and mitochondrial metabolic pathways

For developmental and metabolic processes an effective energy supply is required. Generation of ATP via oxidative phosphorylation is the most efficient way for energy supply [32], [33]. Mitochondria account for 20% of the liver cell volume and are exquisitely sensitive to environmental changes regarding efficiency and accuracy of mitochondrial replication [34]. The observed regulation of the oxidative phosphorylation (OXPHOS) at stage 1 dpn (Figure 2) may be considered as stress response due to a new life situation. The nutrient supply of the neonatal piglets changes from the parenteral route via the umbilical cord and some swallowing of amniotic fluid to enteral nutrition with colostrum containing compounds new to the individual (e.g. fat, lactose, immunoglobulins) [35]. This may cumulate in an altered metabolic status in terms of OXPHOS. The down regulation of genes associated to the mitochondrial respiratory chain suggests a possibly disturbed electron transfer within the OXPHOS. Disturbed mitochondrial oxidative phosphorylation could be of relevance regarding the growth retardation observed for neonates of the HP group compared to the AP group [36]. This might be accompanied by an altered mitochondrial production of reactive oxygen species. The mitochondrial alterations are possibly to be accompanied by a decreased energy production resulting in an altered AMP/ATP-ratio. Transcript levels of the cellular energy sensor AMPK (AMP-dependent protein kinase) showed an increased expression (PRKAA2). AMPK is targeting various genes contributing to growth related signal cascades as well as fuel dependent pathways like fatty acid oxidation or steroid synthesis [37], [38]. The increased expression of PRKAA2 in HP offspring at stage 1 dpn could be part of a compensation process due to the reduced expression of genes involved in the oxidative phosphorylation and a possibly reduced cellular ATP content. Consistently, key enzymes of the energy consuming steroid biosynthesis, HMGCR and DHCR7, showed a decreased expression at stage 1 dpn in HP offspring. A decreased synthesis of important cellular compounds like cholesterol may occur. The transcription factor PGC-1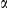 (Peroxisome proliferator-activated receptor 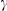 coactivator-1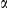) is another downstream, but not directly regulated effector protein of AMPK, which regulates multiple aspects of cellular energy metabolism [39], [40]. Although PGC-1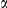 is known to act as a positive regulator on the mitochondrial biogenesis and the expression of ROS-detoxifying enzymes [41], the diet-dependent mRNA expression of PPARGC1A led neither to an adaptation of processes related to mitochondrial dysfunction nor an increased transcription of antioxidative enzymes. This may also indicate the snapshot character of the microarray analysis, because the increased expression of PPARGC1A contributes to a compensatory process, when levels of PRKAA2 as well as OXPHOS associated genes showed an equal mRNA expression at stage 28 dpn. The diet-dependent diametrical alteration of OXPHOS associated genes leads to the conclusion that the ability of porcine offspring to balance metabolic modulation is remarkably high. Because the integrity of mitochondria is seen as an important factor for metabolic health [42]–[44], diet-dependent alterations affecting transcriptional expression patterns of mitochondria related pathways are of interest. Therefore, the susceptibility of OXPHOS to be down regulated as a response to environmental factors (stage 1 dpn) as well as the up regulation of genes associated with mitochondrial dysfunction in HP offspring (stage 188 dpn) may contribute to the development of a predisposition to degenerative diseases in terms of fetal programming after a maternal protein excess. Moreover, at stage 188 dpn in HP offspring a major activator of mitochondrial metabolism, PRKAA1 encoding AMPK, showed diminished expression. This may also contribute to the predisposition to metabolic diseases because AMPK is targeted by antidiabetics [37], [45]. Moreover, the increased expression of genes associated with fatty acid elongation in mitochondria in HP offspring at stage 188 dpn may contribute to mitochondrial alterations. The fatty acid elongation in mitochondria takes place in the mitochondrial matrix and is essentially the reverse of the beta-oxidation by acting primarily on fatty acyl-CoA substrates shorter than 16 carbons. Furthermore, there were clues for a diet-dependent transcriptional alteration of genes associated with the lipid metabolism in HP offspring, including fatty acid metabolism and PPAR signaling. These modulations might contribute to compensatory regulations in terms of growth and body composition. Taken together, these findings suggest that energy sensing pathways and their signals are important in the adaptive response to prenatal nutritional environment.

### Signaling of intracellular metabolism

The mTOR signaling acts as an important nutrient sensing pathway that controls protein synthesis in mammalian cells at the level of translation [46]. Upstream signaling events of mTOR signaling include alterations in amino acid availability, abundance of hormones, AMP and growth factors [47]. Thus, mTOR signaling is involved in regulating individual cell growth, growth performance, and developmental processes [48], [49]. An increase of mTOR signaling from fetal to neonatal stage was observed in AP offspring with no change in the HP group. This may account for a reduced protein synthesis in HP offspring and thus, contribute to the observed growth retardation and altered metabolic status as proposed by Inoki et al. [50]. In addition, mTOR signaling might be also involved in compensatory growth processes. Therefore, the diet-dependent diametrical regulation of mTOR signaling associated genes between juvenile and adult pigs can be interpreted as successful compensation in terms of growth performance. The success of compensatory processes in HP offspring becomes obvious by the fact that their weight was lowered at stages 94 dpc and 1 dpn compared to the AP group, but similar at stage 188 dpn.

It is well documented that IGF-1 signaling pathway plays an essential role in fetal survival and mediates postnatal growth as well as developmental and ageing processes [51]–[53]. Most of the IGF-1 mediated anabolic pleiotropic effects are mediated through association with IGF-1 receptor [54]. With respect to mRNA expression of hepatic IGF-1 signaling the impact of this pathway in terms of growth retardation and possibly compensatory regulations can be estimated. Regarding gestational low protein diets many studies showed an altered IGF-1 signaling [5], [55], [56]. Therefore, it is of interest that mRNA expression of IGF-1 signaling associated genes was regulated due to gestational HP diets, too. When adjacent ontogenetic stages of both experimental groups were compared, only HP offspring showed altered expression profiles regarding IGF-1 signaling. The down regulation of IGF-1 signaling between fetuses and perinatal piglets in HP offspring may underlay the observed low birth weight in HP offspring. Furthermore, the increased mRNA expression of genes associated with IGF-1 signaling after weaning can be considered as compensatory effects in terms of growth and body weight regulation. Interestingly, the up regulation of IGF-1 signaling took place in a time period, when mRNA expression levels of OXPHOS associated genes, and thus probably the energy production, were within a normal range at stage 28 dpn in HP offspring. Since there is an association between the level of growth factor and the sensitivity of cells to this growth factor, the increased expression of IGF-1 signaling at stage 188 dpn may go along with an increase in insulin sensitivity at adult stage. Notably, other studies investigating effects of gestational dietary protein level showed a high insulin responsiveness in early postnatal stages but a lowered one during adulthood [7], [57], [58].

The growth hormone signaling, another anabolic growth factor signaling, regulates metabolic processes including protein synthesis and has important regulatory effects on protein, carbohydrate and lipid metabolism [59]. The mRNA levels of growth hormone signaling related transcripts showed biphasic regulation during the development of HP offspring. Therefore, the observed down regulation of growth hormone signaling associated genes at birth and their up regulation towards juvenile and adult stages correspond with the growth retardation at stages 94 dpc and 1 dpn and the compensatory growth onwards in adulthood.

Glucocorticoids, a major subclass of steroid hormones, regulate a large number of metabolic, behavioural, cardiovascular and immune functions. Their biologic effect is modulated by the glucocorticoid receptor with succeeding direct and indirect interactions of downstream target genes to modulate gene expression. The glucocorticoid receptor signaling terminates stress reactions and mobilizes energy resources for this purpose. The mRNA expression of genes associated with glucocorticoid receptor signaling was shown to be dependent on maternal gestational protein diets [2], [60], [61]. The biphasic regulation of glucocorticoid receptor signaling in HP prenatal, perinatal and juvenile offspring underlines the postulated increased stress response in HP offspring at birth, which may cumulate in the observed disturbances of mitochondrial activity. Furthermore, the diet-dependent regulation of glucocorticoid receptor signaling between juvenile and adult stage and the resulting increased glucocorticoid receptor signaling at stage 188 dpn in HP offspring suggests that HP offspring was in a kind of alarm alert (activated defence) at adult stage.

### Cell maintenance and proliferation

Cell maintenance and proliferation RAN, a member of the Ras family of small GTPases, is a positive key regulator of mitosis [62] and plays a critical role in multiple cellular functions, including nucleocytoplasmic transport, nuclear envelope assembly and spindle formation during the cell cycle [63]. Therefore, the RAN signaling is involved in cellular growth and developmental processes. The diet and stage dependent expression of RAN signaling was partly diametrically regulated along the ontogenetic development (between stages 94 dpc vs. 1 dpn and within stage 1 dpn), which represents adaptation to different prenatal conditions. An almost similar direction of regulation of genes involved in RAN signaling was found at juvenile and adult stages (between stages 28 dpn vs. 188 dpn and within stage 188 dpn), which may account for compensatory processes.

The pathways concerning the metabolisms of purine and pyrimidine are related to a number of processes including nucleotide biosynthesis, degradation and salvage. The down regulation of genes associated with the metabolisms of purine and pyrimidine probably account for a reduced cellular turnover at stage 188 dpn compared to stage 28 dpn in HP offspring. GADD45B, a gene whose transcription is induced in response to multiple environmental and physiological stress factors, is involved in DNA repair, apoptosis, cell survival and growth arrest [64]. The mRNA expression of GADD45B was increased in HP offspring at stage 188 dpn, which suggests a metabolic priority for cell survival and a terminated compensatory growth performance at adult stage.

Valine, leucine and isoleucine are indispensable branched-chain amino acids. The catabolism of all three amino acids shares the same enzymes during the first steps, including transamination and decarboxylation. As final result, three different CoA derivates are produced, which can be directed towards the synthesis of steroids or ketone bodies and to the citrate cycle. The down regulation of genes associated with the valine, leucine and isoleucine degradation in HP offspring at stages 1 dpn and 28 dpn may correspond to a higher demand for these amino acids than in AP offspring. However, HP offspring increased the expression of genes involved in the degradation of valine, leucine and isoleucine during post weaning development which resulted in an unaltered expression level at stage 188 dpn between the dietary groups.

### Conclusion

Gestational HP diets affected the hepatic expression profiles at prenatal and postnatal stages. The effects encompass a bias of the genome leading to altered responsiveness of energy and nutrient sensing pathways. Obviously the programming of the genomes warrants the adaptations and compensatory growth, but probably at the expense of the predisposition for metabolic disturbances up to adult stage. In order to test this hypothesis the porcine model could be used in an experiment where offspring of sows fed different gestational diets is also objected to different dietary challenges at postnatal stages.

## Materials and Methods

### Animals and sample collection

**Figure 3:**
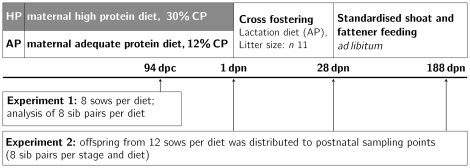
Experimental design.

Animal care and tissue collection procedures followed the guidelines of the German Law of Animal Protection and the experimental protocol was approved by the Animal Care Committee of the State Mecklenburg-Vorpommern (Landesamt für Landwirtschaft, Lebensmittelsicherheit und Fischerei, Mecklenburg - Vorpommern, Germany; LVL MV/TSD/7221.3-1.1-006/04; LALLF M-V/TSD/7221.3-1.2-05/06; LALLF M-V/TSD/7221.3-1.2-013/06). The animal experiment was performed as described [14]. A high protein diet (HP) containing 30% (w/w) crude protein or an adequate protein diet (AP) containing 12% crude protein were formulated to be isocaloric (13.6 MJ ME/kg on average). The gilts of both groups consumed 2.6 kg/d resulting in a significantly different protein intake but equal energy intake [14]. The gestation diets did not affect reproductive parameters like litter size and litter weight as well as percentage of stillborn and mummies [14]. At insemination German Landrace primiparous sows (n = 48) were randomly assigned to either the HP or the AP group (Figure 3). Tissue sampling included offspring of these sows at one prenatal (94 dies post conceptionem (dpc)) and three postnatal time points (1, 28, 188 dies post natum (dpn)). At d 94 of gestation, a subset of eight sows per dietary group was subjected to Caesarean section (EXP1). Sows were anesthetized as described [14]. This experiment was performed over 5 replicates. Eight viable foetuses per sow were collected starting at the tip of the left uterus horn and alternating between left and right horn. Fetuses were killed by i.v. injection of T61 (Intervet, Unterschleissheim, Germany) in the V. cava cranialis and liver samples were immediately collected (approximately 500 mg), frozen in liquid nitrogen, and stored at 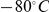 until analysis. Fetuses originated from litters of at least 11 viable piglets. Fetuses of HP fed dams showed a decreased weight compared to AP fetuses at 94 dpc (HP: 622
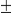
119 g, and AP: 711
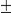
118 g, respectively; 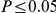; n = 32). The smallest and the heaviest fetus were selected for transcriptome analysis. In the second experiment (EXP2) offspring selected for the postnatal time points was born to primiparous sows after prostaglandin induction of parturition as described [14] and farrowed after a mean pregnancy duration of 115 days. This experiment was conducted over 8 replicates and offspring of a subset of 4 sows (2 per diet per replicate) with a minimum of 11 live born piglets (median litter size = 13) was used. At birth 10 piglets in each litter were distributed over three time points (1, 28, 188 dpn). For the microarray analyses, 8 sib pairs, which were balanced for sex (all stages) and discordant for weight (light and heavy piglet; stages 94 dpc and 1 dpn only) were chosen per stage and diet. Thirty-six hours after birth, the lightest and the heaviest piglet within one litter were killed by i.m. injection of 1.25 mg propionyl-promazine (0.2 ml Combelen, Bayer AG, Leverkusen, Germany) and 50 mg ketamine (Ursotamin, Serumwerk Bernburg AG, Germany). Samples were immediately collected from lobus sinister hepaticus (approximately 500 mg), frozen in liquid nitrogen, and stored at 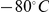 until analysis. The remaining piglets were cross-fostered to non-experimental sows of 2nd to 4th parities, which were on the AP diet during gestation. All sows were fed AP lactation diets. Litter size during suckling was standardized to 11 piglets per sow. Male piglets were castrated at d 4 of age. From weaning (28 dpn) to slaughter (188 dpn), all piglets were individually reared. They had free access to standard diets formulated for post-weaning (d 29 to d 76), growing (d 77 to d 105) and finishing periods [65] and had the same feed intake. At 28 dpn and 188 dpn of age, pigs were weighed after an overnight fast and killed by electronarcosis followed by exsanguination in the experimental slaughterhouse of FBN. Liver tissue was immediately collected from lobus sinister hepaticus, frozen in liquid nitrogen, and stored at 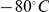 until use for RNA isolation. In our experiment piglets (1 dpn) born from HP sows had significantly lower birth weight (HP: 1.21
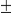
0.04 kg, and AP: 1.41
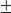
0.04 kg respectively, 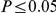) that mainly resulted from reduced body fat, whereas body mass index and ponderal index did not differ from the AP group [14]. Mean birth weights of animals of the HP group used for microarray analyses were numerically but not significantly lower than birth weights of AP offspring (HP: 1.31
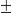
0.31 kg, and AP: 1.36
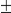
0.31 kg, respectively; 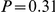; n = 32). At 28 dpn, the animals of the AP and HP group showed no difference in body weight (HP: 7.36
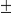
1.54 kg, and AP: 7.59
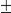
2.14 kg respectively; 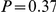; n = 32) and body composition, i.e. muscle, fat depots, bones, skin, as well as in analytical components (protein, fat, ash, moisture) or structural and biochemical properties of fat and muscle tissues [25]. Also at 188 dpn offspring of AP and HP sows did not differ significantly in body weight (HP: 131.41
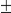
7.11 kg, and AP: 131.55
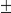
15.11 kg respectively; 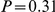; n = 32). Like at earlier ages animals of the HP group tended have lower meat but higher fat percentage, but the differences were not significant [24].

### RNA isolation, target preparation and hybridization

According to the manufacturer's protocol total RNA from individual liver samples was isolated using Tri-Reagent (Sigma-Aldrich, Taufkirchen, Germany). After DNase treatment a column-based purification using the RNeasy Mini Kit (Qiagen, Hilden, Germany) was done. The RNA samples were visualized on 1% agarose gels containing ethidium bromide to check RNA integrity. RNA was quantified by spectrometry with a NanoDrop ND-1000 spectrophotometer (PEQLAB, Erlangen, Germany). To ensure the absence of a DNA contamination within the isolated RNA a PCR amplification with the porcine glyceraldehydes-3- phosphate dehydrogenase (GAPDH) gene was done (Forward primer: AAGCAGGGATGATGTTCTGG; Reverse primer: ATGCCTCCTGTACCACCAAC). All RNA samples were stored at 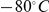 until downstream analysis was performed. For the microarray experiments individual biotin-labeled cRNA was synthesized by the Gene Chip 3 Express Kit (Affymetrix, Santa Clara, CA, USA). The cRNA was fragmented (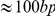) and hybridized on Affymetrix GeneChip porcine Genome Array. After staining and washing steps the arrays were scanned (Affymetrix, Santa Clara, CA, USA).

### Data analysis

The programming was done in R using Bioconductor [66]. Firstly, a quality control was performed. At 94 dpc 15 AP-samples met the appropriate quality control criteria (94 dpc-HP: 16; 1 dpn-AP: 15; 1 dpn-HP: 16; 28 dpn-AP: 14; 28 dpn-HP: 15; 188 dpn-AP: 16; 188 dpn-HP: 16 samples). Samples were GCRMA normalized (Log2). The MAS5 algorithm was used to skip those transcripts which were expressed in less than 50% of the animals within one dietary group per stage. For a second filtering step standard deviations were calculated for each probe-set over all subsets of arrays of the particular comparisons. Probe-sets with a low standard deviation (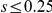) were discarded, because such transcripts are not likely to be regulated. Relative changes in mRNA levels were determined using a mixed model analysis, including effects of dietary treatment, sex, mother, weight (for stages 94 dpc and 1 dpn) and interaction between sex and dietary treatment (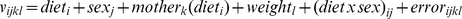). P-values (significance set at 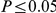) for each stage were converted to a set of q-values (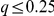) using the algorithm proposed by Storey and Tibshirani [67]. In general, results are given for the comparisons in the direction of HP vs. AP; thus ‘up regulation’ or ‘increased expression’ indicates higher expression in HP than in AP. Analysis of the pathways involved was carried out using Ingenuity Pathway Analysis [68]. The up-to-date annotation of Affymetrix probe-sets to EnsEMBL Sscofa 9 was used. The Affymetrix GeneChip Porcine Genome Array contains 24,123 probe-sets that interrogate 20,689 transcripts that were assigned to known genes [26]. All the microarray data is MIAME compliant and the raw data has been deposited in a MIAME compliant database, the National Center for Biotechnology Information Gene Expression Omnibus (www.ncbi.nlm.nih.gov/geo) (accession numbers: GSE25482 and GSE25483).

### Pathway analysis

Gene lists from microarray results were submitted to the manually curated database ‘Ingenuity Pathways Analysis’ to elucidate putative pathways associated with an altered gene expression in porcine liver. The focus was on those canonical pathways which came up at least once within the top ten regulated pathways within one single analysis. It should be noted here that the interactions presented in the networks are not specific for porcine liver tissue, as the database contains literature from many different research areas. The web server named DiRE (for predicting distant regulatory elements; access at http://dire.dcode.org; [69]) was used to determine common transcription factor binding sites (TFBS) of the diet-dependent regulated genes.

### Quantitative real-time RT-PCR

First-strand cDNA was synthesized from 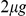 of total RNA (n = 14 per diet and stage) using random primers and oligo d(T) 13VN in the presence of Superscript II reverse transcriptase (Invitrogen, Karlsruhe, Germany). In order to survey expression of the liver tissue samples, total transcript levels of selected target and reference genes (Table 4) were quantified by real-time quantitative PCR (qPCR) performed on a LightCycler 480 system using the LightCycler 480 SYBR Green I Master (Roche, Mannheim, Germany). The amplification was conducted in duplicate according to manufacturer's instructions using 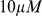 of each primer. Reactions were performed in a final volume of 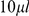 using 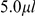 of LightCycler 480 SYBR Green I Master (Roche), 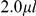 of Aqua dest., 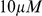 (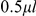) of each primer (Table 4) and 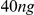 (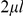) cDNA. The temperature profiles comprised an initial denaturation step at 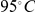 for 10 min and 40 cycles consisting of denaturation at 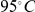 for 15 s, annealing at 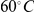 for 10 s and extension/fluorescence acquisition at 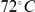 for 15 s. For all the assays threshold cycles were converted to copy numbers using a standard curve generated by amplifying serial dilutions of an external PCR standard (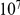
−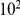 copies). At the completion of the amplification protocol, all samples were subjected to melting curve analyses and gel electrophoresis to verify the absence of any non-specific product. To account for variation in RNA input and effciency of reverse transcription the calculated mRNA copy numbers were normalized by dividing with a normalization factor derived from the expression of the reference gene. In total, 28 individual liver mRNA samples were analyzed in duplicate per stage. Data were analyzed using the PROC MIXED, including effects of treatment, sex, mother, birth weight and interaction between sex and treatment (SAS version 9.1; SAS Institute, Cary, NC). Differences were considered significant at 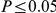.

**Table 4:** Primer used to verify microarray experiments in liver tissue by qRT-PCR.

Gene name	Probe set ID	Sequence 5′ – 3′	Accession no.	T(  C)	Size (bp)
DHCR7	Ssc.5455.1.S1_at	For GCATGACACTGACTTCTTCTC Rev CCCACCTCCACTTTATTC	BE232966	60	136
GADD45B	Ssc.14764.1.A1_at	For GGACTTAGACTTTGGGACTTG Rev GTAAGCCTCCCATCTCTCTT	BF708594	60	140
HMGCR	Ssc.16088.1.S1_at	For GTGCTGGTCTGTTTTGATTT Rev TGCAGTGATTTGTTTTCTTG	BP436947	60	159
NDUFA 4	Ssc.7315.1.S1_at	For TCCTGCTTAGTCCCCGACCTT Rev ACAGTGCTGCTCCAGTACCTCC	CF793329	60	164
NDUFS 7	Ssc.1681.1.S1_at	For CGGCTACTACCACTACTCCT Rev ATCCGCAGTCTCTTCTCC	CK455535	60	156
NDUFS 8	Ssc.2312.1.S1_at	For AGTTTGTGAACATGCGTGAG Rev CTCAAATGGGTAGTTGATGG	BI181006	60	152
PPARGC1A	Ssc.16864.1.S1_at	For GTAAATCTGCGGGATGATGG Rev TGGTGGAAGCAGGATCAAAG	AB106108	60	208
PRKAA 1	Ssc.8107.1.A1_at	For TTGTTAATTTCATAAACTTTGCTTC Rev GTGCAGCCTTGACATACTC	BF712533	60	193
PRKAA 2	Ssc.16257.1.S1_at	For TCTGTAATTCTGTTTTGCCTACG Rev AGCAAGAAGGTGATGCCAAG	NM214266	60	168
RPL 10*	Ssc.9130.1.A1_at	For CTGTGTTCGTCTTTTCTTCC Rev TCATCCACTTTTGCCTTCT	BI181297	60	199

DHCR7 - 7-dehydrocholesterol reductase; GADD45B - growth arrest and DNA-damage-inducible, beta; HMGCR - 3-hydroxy-3-methylglutaryl-coenzyme A reductase; NDUFA4 - NADH-ubiquinone oxidoreductase MLRQ subunit; NDUFS7 - NADH-ubiquinone oxidoreductase 20 kDa subunit; NDUFS8 - NADH-ubiquinone oxidoreductase 23 kDa subunit; PPARGC1A - Peroxisome proliferator activated receptor gamma coactivator 1 alpha; PRKAA1 – 5′-AMP-activated protein kinase, catalytic alpha-1 chain; PRKAA2 – 5′-AMP-activated protein kinase, catalytic alpha-2 chain; RPL10 - Ribosomal protein 10.

*House keeping gene.

## Supporting Information

Table S1 Significantly regulated probe-sets due sex at various stages.
(XLS)
